# Shining the PSMA spotlight on peritoneal metastases in prostate cancer

**DOI:** 10.1007/s00259-025-07703-3

**Published:** 2025-12-11

**Authors:** Jonathan Kuten, Stephanie Chahwan, Charlie White, Audrey Mauguen, Heiko Schöder, Simone Krebs

**Affiliations:** 1 Molecular Imaging and Therapy Service, Department of Radiology, Memorial Sloan Kettering Cancer Center, 1275 York Avenue, New York, NY 10065 USA; 2 Department of Epidemiology & Biostatistics, Memorial Sloan Kettering Cancer Center, New York, NY USA; 3 Department of Nuclear Medicine, The University of Texas MD Anderson Cancer Center, Houston, TX USA; 4 Department of Imaging Physics, The University of Texas MD Anderson Cancer Center, Houston, TX USA; 5 Institute of Data Science in Oncology, The University of Texas MD Anderson Cancer Center, Houston, TX USA

**Keywords:** Prostate cancer, Peritoneal metastases, PSMA PET/CT, FDG PET/CT, ^177^Lu-PSMA-617

## Abstract

**Purpose:**

This study aimed to retrospectively characterize patients with biochemical failure and peritoneal metastases detected by PSMA PET/CT, describe the detectability of ^18^F-FDG PET/CT and conventional imaging; and assess patient outcome.

**Methods:**

We searched an institutional database for patients who underwent ^68^Ga-PSMA-11 or ^18^ F-DCFPyL PET/CT between 5/2016 and 9/2024 and measured the SUVmax of the most avid peritoneal lesion. We assessed correlative CT, MRI, or FDG PET/CT performed within 90 days and validated peritoneal metastases via composite reference. Survival was estimated with Kaplan-Meier analysis. Seven patients subsequently treated with ^177^Lu-PSMA-617 were analyzed.

**Results:**

Of 9,877 screened patients, peritoneal metastases were confirmed in 57. Most had Gleason ≥ 4 + 3 (81%) and median PSA was 10.0 ng/ml (range 0.34–1,304); 54% were castration-sensitive. Isolated peritoneal disease occurred in 19 (33%). The median SUVmax was 8.2. Among 29 patients with correlative CT/MRI, 41% were negative. Of 14 patients with correlative FDG PET/CT; 43% demonstrated FDG-avid peritoneal disease. On follow-up (*n* = 42), peritoneal disease improved in 38%. Of those with isolated peritoneal metastases and follow-up data (*n* = 12), 58% improved. Median overall survival from detection was 53 months (95% CI: 29–not reached), and associated with lower PSA, fewer peritoneal lesions, and non-castration resistance. All seven patients treated with ^177^Lu-PSMA-617 progressed.

**Conclusion:**

Peritoneal metastases from prostate cancer are rare. Nevertheless, PSMA-avid peritoneal lesions may represent metastatic spread from prostate cancer, even in the absence of systemic disease. FDG PET/CT and CT/MRI often miss peritoneal metastases. Peritoneal disease does not necessarily indicate poor prognosis, particularly when isolated.

**Graphical abstract:**

**Figure:**
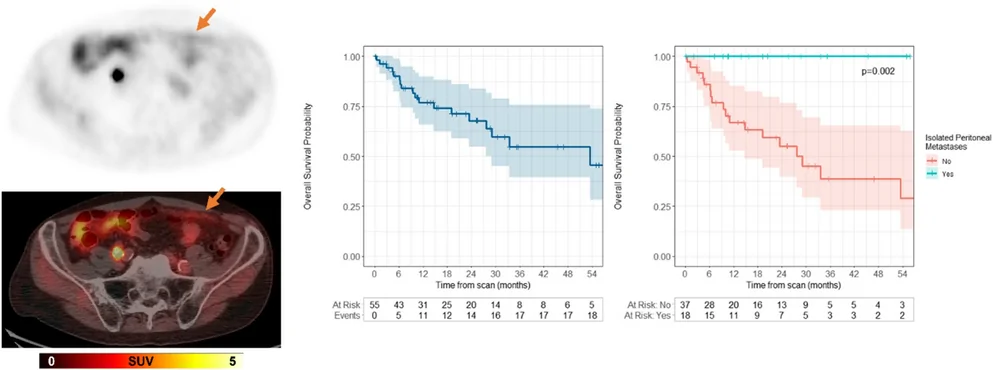

**Supplementary Information:**

The online version contains supplementary material available at 10.1007/s00259-025-07703-3.

## Introduction

Prostate cancer is the most common cancer in men in the US, with 299,010 new cases diagnosed in 2024, accounting for 29% of all male cancers. Its incidence has increased by 3% per year since 2014. It is also the second leading cause of cancer death among men, with 35,250 deaths in 2024 [1]. Peritoneal metastases from prostate cancer are considered rare—only 1–7% of metastatic sites per autopsy studies [2, 3]. A limited number of case reports in the literature described peritoneal metastases from prostate cancer, with some being isolated sites of disease [4–18]. Prostate cancer cells may spread to the peritoneum by either direct invasion from the primary tumor, iatrogenic seeding after prostate surgery, or metastatic spread—the latter previously regarded as more common in advanced poorly differentiated tumors or tumors with neuroendocrine differentiation [6, 11, 19, 20], which are associated with poor prognosis.

Several studies have shown the superior sensitivity and accuracy of prostate-specific membrane antigen (PSMA) PET/CT over conventional imaging for evaluating the extent of disease in patients with prostate cancer [20]. With the introduction of highly sensitive PSMA-targeting molecular imaging, one might expect the incidence of unusual sites of metastatic disease to rise, including peritoneal disease, as a result of better detection.

Based on clinical observations, we initiated this retrospective study to evaluate the characteristics and clinical outcome of patients with biochemical failure (BCF) who were referred for PSMA PET and were found to have peritoneal metastases. We also assessed the detectability of peritoneal disease by ^18^F-FDG (FDG) PET/CT and conventional imaging. A sub-analysis was performed in patients treated with ^177^Lu-PSMA-617 radioligand therapy (RLT). We hypothesized that peritoneal metastases in BCF prostate cancer, though rare, can be identified on PSMA PET/CT, and characterization of their clinical features, outcomes, and treatment response may provide valuable insights.

## Materials and methods

### Patient selection

We conducted a DataLine search for all ^68^Ga-PSMA-11 and ^18^F-DCFPyL (^18^F-piflufolastat) PET/CT studies performed for BCF between May 2016 and September 2024 in which the clinical imaging report described peritoneal metastases. This retrospective study was approved by the institutional review board, and the need for written informed consent was waived. Patients with peritoneal metastases from other coexisting cancers, duplicates, and those with spread only by direct extent from the primary tumor were excluded. All scans were reviewed by three of the authors (JK, SC, SK) to ensure that only true peritoneal disease would be included. Peritoneal metastases were validated using composite reference, including histopathology, follow-up imaging, or consensus read when follow-up and pathology were not available. Correlative CT/MRI or FDG PET/CT done within 90 days of the PSMA scan were also reviewed.

^68^Ga-PSMA-11 and ^18^F-DCFPyL were performed as per standard protocol in line with current guidelines, on various state-of-the-art PET scanners by GE or Siemens. Correlative CT or MRI were contrast-enhanced (detailed imaging protocols can be found in the supplemental materials).

Maximum standardized uptake value (SUVmax) of the most avid peritoneal lesion was recorded in all PSMA and FDG studies (the highest SUV was recorded if several lesions were found). We also recorded the number of lesions (few < 5 versus multiple ≥ 5), morphology (subcentimeter lesions regarded as nodularity versus implants ≥ 1 cm, or both), and other sites of disease.

For a subgroup of patients who went on to receive ^177^Lu-PSMA-617 therapy at our institution, we evaluated their clinical and imaging data to assess response to treatment and tolerability.

### Statistical analysis

Continuous variables were summarized using median, interquartile range (IQR), and range, and were compared between groups using Wilcoxon and Kruskal-Wallis rank sum tests. Categorical variables were summarized using frequency and percentage and compared between groups using Fisher’s exact test. Spearman’s correlation was used to evaluate the relationship between PSMA uptake and PSA level or FDG uptake. Overall survival (OS) was estimated using the Kaplan-Meier estimator and was defined as time from PSMA scan to death. Patients who were alive were censored at the last follow-up. Median follow-up was estimated using the reverse Kaplan-Meier method. The log-rank test was used to compare OS between groups. A p-value < 0.05 was considered statistically significant.

## Results

**Fig. 1:**
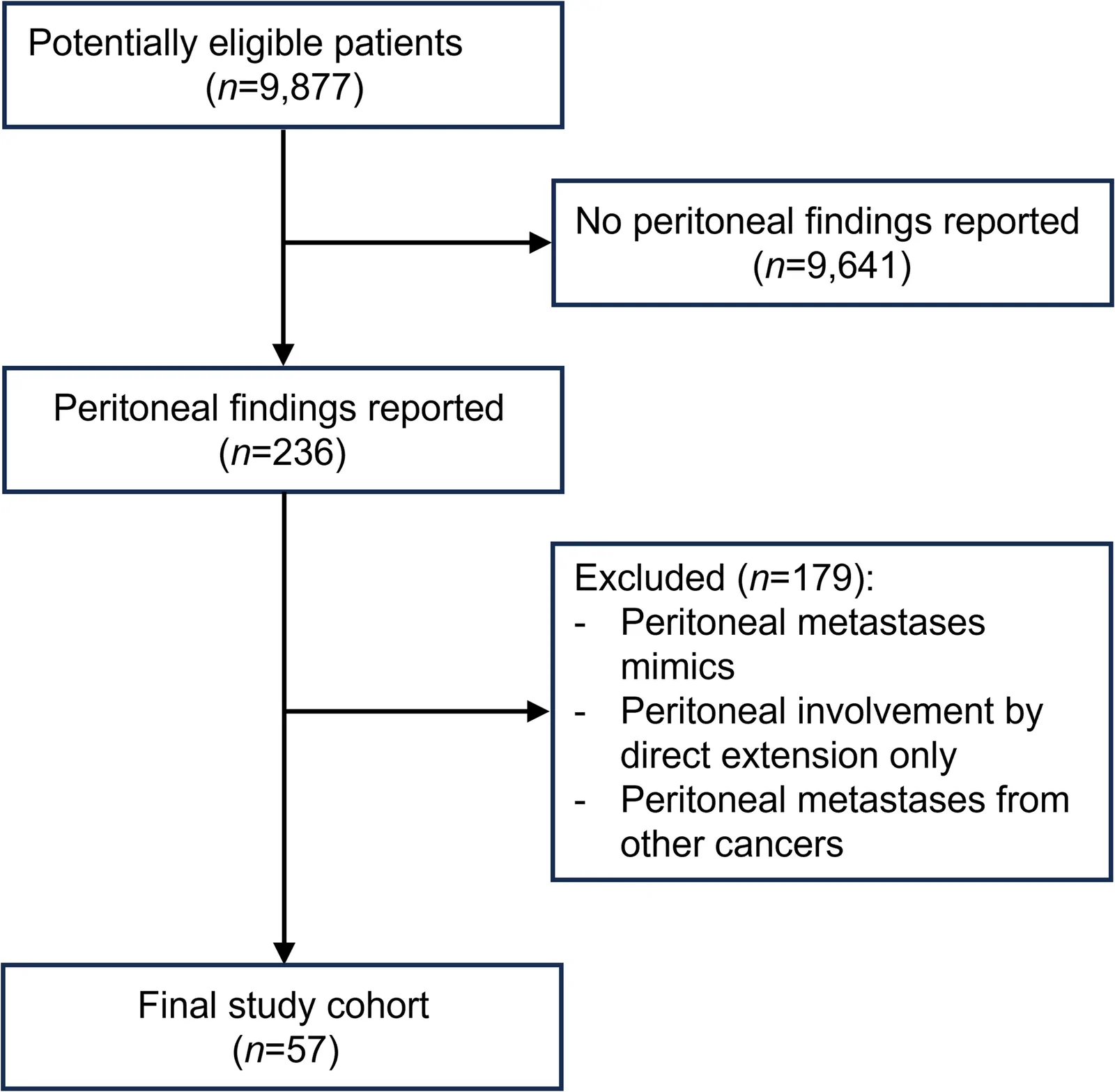
Consolidated Standards of Reporting Trials (CONSORT) diagram

A cohort of 57 patients with BCF and peritoneal metastases was retrieved out of 9,877 patients who underwent PSMA imaging for staging or restaging of prostate cancer (0.5% of patients) (Fig. 1; Table 1). Overall, 42/57 patients (74%) had ^68^Ga-PSMA-11 scans (one had PET/MRI), 14 patients had ^18^F-piflufolastat (Pylarify) scans, and ^18^F-rhPSMA-7.3 was used for 1 patient (Table 2). SUVmax and tumor-to-liver uptake ratio did not differ significantly between Ga-PSMA-11 and Pylarify (all *p* = 0.3). Peritoneal metastases were confirmed by pathology in 17 patients (30%), follow-up imaging in 31 (54%), and consensus read in 9 (16%). In these patients, 81% (43/53) had a Gleason score of 4 + 3 or higher, median PSA was 10.01 ng/ml (range: 0.34-1,303.82), and 54% of patients were castration-sensitive. Prior prostatectomy was noted in 38 patients (67%). Forty-two patients (74%) were previously treated with radiation therapy to the prostate either as initial treatment or salvage. A full list of prior and current treatments is available in Table S1.

**Table 1:** Patient characteristics

Characteristic	*N* = 57^1^
Age at scan	
Median [Range]	71 [56, 93]
Gleason Score	
3 + 4 and below	10 (19%)
4 + 3 and above	43 (81%)
Unknown	4
PSA ng/ml	
Median (IQR)	10.01 (1.83, 37.39)
[Range]	[0.34, 1,303.82]
Castrate Resistant	
No	31 (54%)
Yes	26 (46%)
Peritoneal metastases Validation	
Biopsy	17 (30%)
Imaging	31 (54%)
None	9 (16%)
Prior Prostatectomy	38 (67%)
Prior Prostate RT	42 (74%)
Other sites of metastases	
None	19 (33%)
Nodal	34 (60%)
Osseous	22 (39%)
Lung	8 (14%)
Liver	5 (8.8%)
Pleural	4 (7.0%)

^1^n (%)

**Table 2:** PSMA scan characteristics

Characteristic	*N* = 57^1^
Tracer used	
^18^F-rhPSMA-7.3	1 (1.8%)
^68^Ga-PSMA-11	42 (74%)
^18^F-Piflufolastat	14 (25%)
Peritoneal PSMA SUVmax	
Median (IQR)	8.20 (5.30, 16.20)
[Range]	[1.30, 67.00]
Peritoneal metastases Number	
< 5	41 (72%)
≥ 5	16 (28%)
Peritoneal metastases Morphology	
≥ 1 centimeter	19 (33%)
< 1 centimeter	23 (40%)
Both	15 (26%)

^1^n (%)

Forty-one patients (72%) had few peritoneal metastases (< 5) and 16 (28%) had multiple (≥ 5). Peritoneal metastases were subcentimeter in 23 patients (40%), at least 1 centimeter in 19 (33%), and a combination of both in the rest (26%). Median SUVmax in peritoneal lesions was 8.2 (range: 1.3–67.0), significantly lower in subcentimeter nodules than in larger implants (median SUVmax 5.1 vs. 13.6, respectively, *p* < 0.001). Castration resistance was marginally associated with higher PSMA SUVmax versus non-castration-resistant, though this was not statistically significant (11.8 vs. 7.7, *p* = 0.055).

**Fig. 2:**
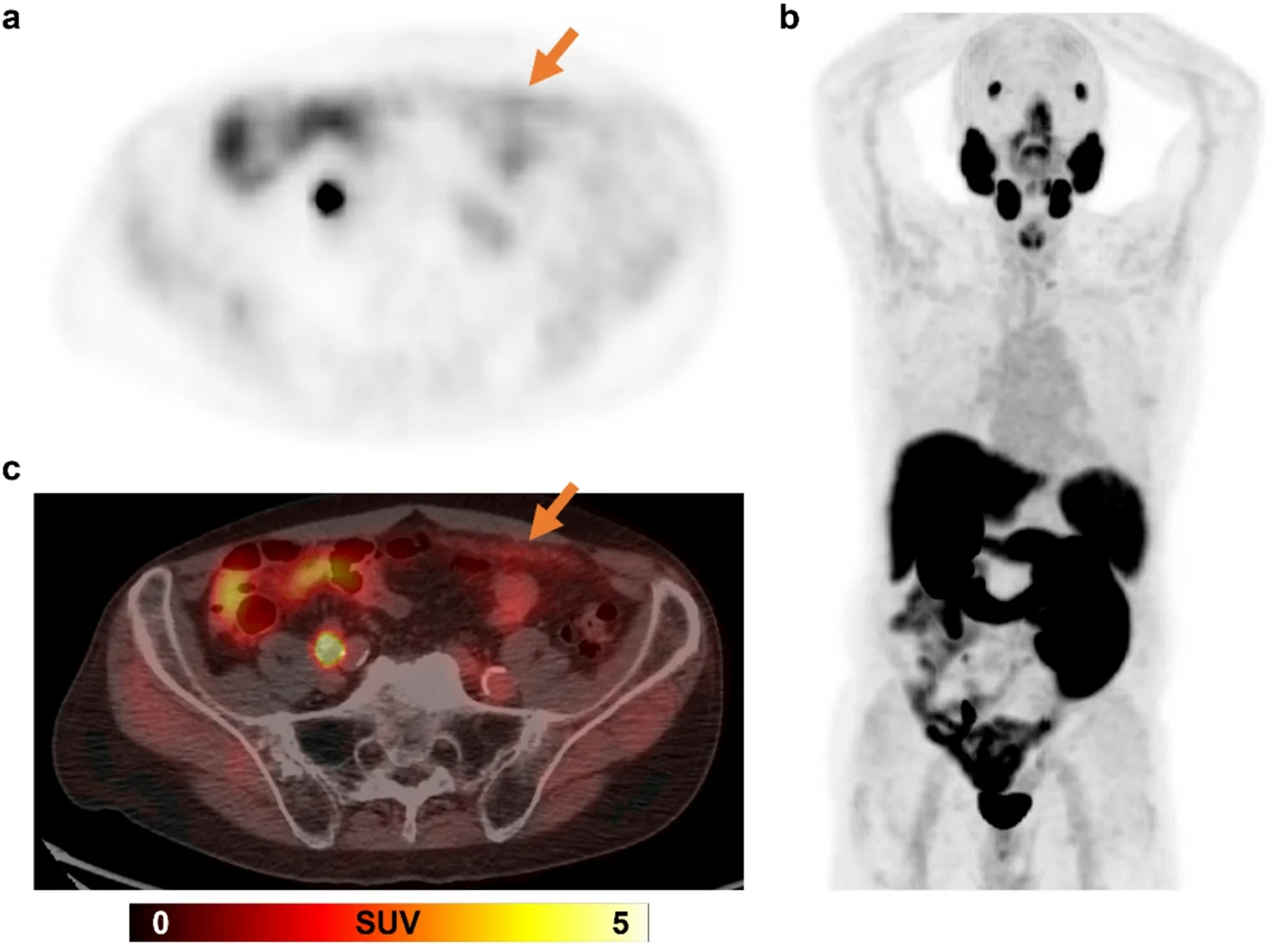
Patient with isolated PSMA-avid peritoneal metastases. 81-year-old man with prostate adenocarcinoma (Gleason 3 + 4) post-prostatectomy and intermittent ADT, with rising PSA (6.63 ng/mL). (**a**) Axial ^68^Ga-PSMA PET image shows linear avidity (SUVmax 2.5; arrow) in the left anterior lower quadrant, corresponding to omental nodularity (arrow) on axial fused PET/CT (**b**), without other sites of disease seen on maximum-intensity projection image (**c**)

Gleason score, the number of peritoneal lesions, and FDG avidity did not corelate with PSMA SUVmax in peritoneal disease. Isolated peritoneal metastases (Fig. 2a, b, c) were found in 19 patients (33%) and other patients had at least one more site of metastatic spread, most often nodal disease (60%), followed by bone (39%), lung (14%), liver (9%), and pleura (7%). Only 1 (3%) and 7 (32%) patients had numerous nodal or bone metastases, respectively—defined as more than 20 metastases.

Two patients had biopsy-proven neuroendocrine differentiation of their disease, one of whom had correlative FDG PET/CT, with the highest recorded FDG SUVmax (18.7) in the cohort. Two patients had associated small volume ascites, non-PSMA-avid.

### Correlative imaging

Twenty-nine patients had correlative CT/MRI performed within 90 days of PSMA PET imaging (median − 21 days, range [-81, 7]). Of these, 17 (59%) were concordant with PSMA PET for the presence of peritoneal metastases. Twelve correlative scans were read as negative for peritoneal metastases, with 38% being falsely negative upon retrospective review. One patient had a true-negative CT 47 days prior to PSMA PET. Of 14 patients with correlative FDG PET/CT (performed within a median of 16 days from PSMA PET, range [-64, 79]), only 6 (43%) had FDG-avid peritoneal disease and the remainder were non-FDG-avid. Among those with FDG-avid peritoneal disease, median FDG SUVmax was 9.0 (range: 3.2–18.7). PSMA and FDG SUVmax in peritoneal disease were positively correlated (0.66, *p* = 0.024).

### Follow-up

**Fig. 3:**
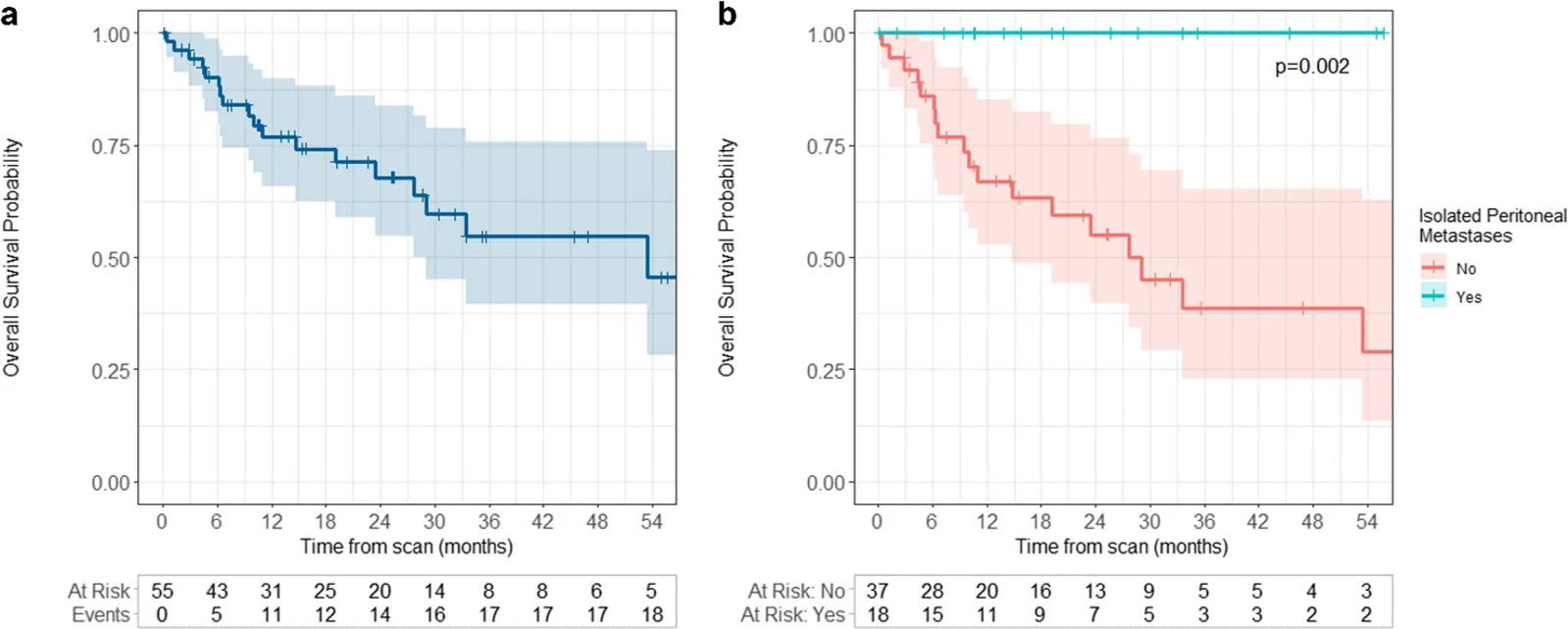
Overall survival (Kaplan-Meier) curves. Kaplan–Meier overall survival curves of all patients (**a**) and stratified by presence of isolated peritoneal metastases (**b**)

Forty-two patients had follow-up imaging available for review. Of these, 19 patients (45%) had progression of peritoneal disease; 7 (17%) had stable disease, and 16 (38%) had decreased/resolved disease. Among those with isolated peritoneal metastases (*n* = 12), 58% showed improved disease. When peritoneal metastases progressed, so did the overall disease burden. Eleven of 16 patients (69%) with improved or resolved peritoneal disease were treated with ADT and ARPI. Other treatments given to patients in the present cohort were stand-alone or combinations of chemotherapy, radiation therapy, surgery, ^177^Lu-PSMA-617 RLT, PARPi, or surveillance. Median follow-up period (since peritoneal disease detection on PSMA imaging) was 25 months (95% CI 16, 35). Two patients did not have follow-up data and were excluded from survival analysis. The median OS from detection of peritoneal metastases was 53 months (95% CI 29, Not Reached). None of the patients who had only peritoneal metastases perished during follow-up (Fig. 3a, b). Patients with < 5 peritoneal metastases, PSA < 10, and those who were castrate-sensitive had better OS (all *p* < 0.05). We did not detect a difference in OS by Gleason score (*p* = 0.7) or peritoneal metastases size (*p* = 0.09).

### ^177^Lu-PSMA-617 RLT

**Fig. 4:**
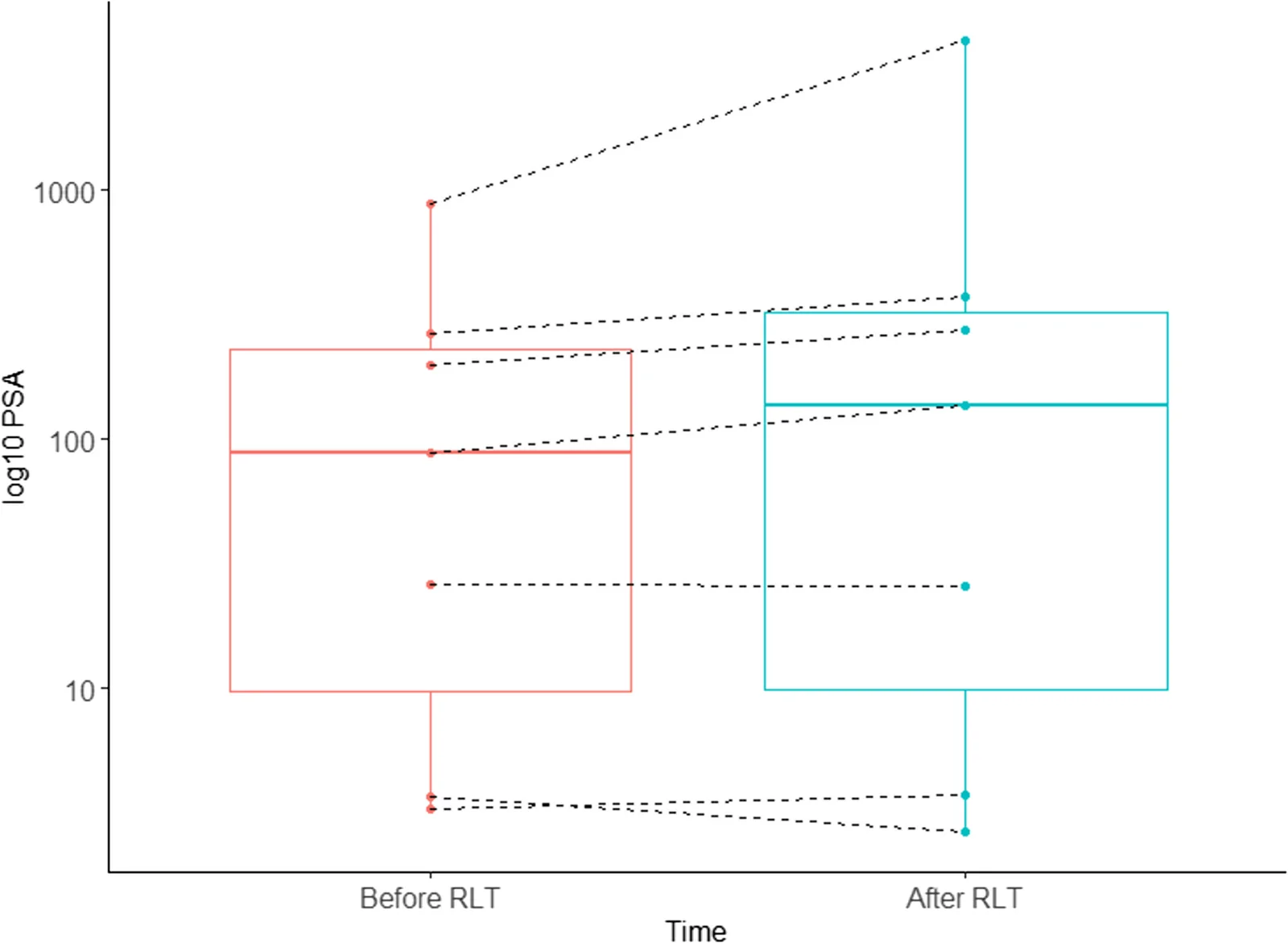
PSA at baseline and after treatment with ^177^Lu-PSMA-617

Seven patients went on to receive ^177^Lu-PSMA-617 RLT at our institution. All patients had at least one additional site of metastatic disease aside from the peritoneum and all were previously treated with multiple lines of treatment including chemotherapy. All 7 patients had progression of disease, as determined per multidisciplinary team discussion based on PSA levels and clinical evaluation, as well as imaging when it was available. Only two patients received all six prescribed cycles of treatment; the other patients ended their therapy due to disease progression. The median cumulative dose was 22,533 MBq (range: 200-1,198). Treatment was tolerated well with no need for dose modification or treatment postponement. None of the patients suffered bowel obstruction. Median PSA level increased from pre- (88.95 ng/ml, range: 3.28, 887.26) to post-therapy (136.84 mg/ml, range: 2.65, 3,957.37; Fig. 4). Four patients had follow-up PSMA PET/CT; 3 had overall radiologic progression of disease, and none had decreased disease in the peritoneum. One patient had mixed responses on his interim PSMA PET/CT including some decreased peritoneal lesions but showed progression of disease at all locations on his end-of-treatment PSMA PET/CT.

Additionally, 5 patients were reportedly treated with ^177^Lu-PSMA-617 RLT at outside institutions, for whom we only have limited data. Per clinical notes, one had progression of disease, one had “mixed response” including decreased peritoneal disease, one had resolved disease after two cycles, and one had “good response” after six cycles. Characteristics of patients treated at our institution with ^177^Lu-PSMA-617 RLT are summarized in Table 3.

**Table 3:** ^177^Lu-PSMA-617 treatment

Characteristic	*N* = 7^1^
Treatment Outcome	
POD	7 (100%)
Number of Cycles	
1	1 (14%)
3	3 (43%)
4	1 (14%)
6	2 (29%)
Total Dose (MBq)	
Median (IQR)	22521.9 (21539.7, 36504.2)
[Range]	[7414.8-44322.0]
Anemia by grade	
0	2 (29%)
1	3 (43%)
2	2 (29%)
Thrombocytopenia by grade	
0	4 (57%)
1	2 (29%)
2	1 (14%)
Lymphopenia by grade	
0	2 (29%)
1	2 (29%)
2	2 (29%)
3	1 (14%)
Renal Toxicity	0 (0%)
Liver Toxicity	0 (0%)
Dose Reductions	0 (0%)
Baseline PSA	
Median (IQR)	88.95 (14.9, 232.1)
[Range]	[3.2, 887.2]
PSA (end of treatment)	
Median (IQR)	136.8 (14.8, 324.9)
[Range]	[2.7, 3,957.4]

^1^n (%)

### Pitfalls

During data collection, we encountered a multitude of peritoneal findings on PSMA PET scans that are considered potential mimics of peritoneal metastases from prostate cancer. These can be divided into PSMA-avid and non-avid findings (Table 4). The most common mimic in the current cohort was splenosis.

**Table 4:** Mimics of peritoneal metastases from prostate cancer

PSMA non-avid findings (*n* = 14)	PSMA-avid findings (*n* = 8)
Indeterminate peritoneal nodularity, stable for years and likely benign (3)	Splenosis (4)
Lymphocele / inclusion cyst / unspecified cystic lesion (2)	Melanoma metastases (higher FDG avidity) (1)
FDG-avid mesenteric mass with calcifications, likely carcinoid or sclerosing mesenteritis (1)	Colon cancer metastases, mildly PSMA-avid (1)
Colon cancer metastases (2)	Distal ileal uptake, inflammatory per biopsy (1)
Neuroendocrine tumor metastases, DOTATATE avid (2)	Renal cell carcinoma metastases (FDG and PSMA avid) (1)
Diffuse peritoneal haziness with small-volume ascites, likely inflammatory/infectious (e.g. pancreatitis) (1)	
Post-appendicitis inflammatory findings (1)	
Appendiceal mucinous tumor metastases (1)	
Gastrointestinal stromal tumor (1)	

Numbers in parentheses indicate the number of patients with diagnosis

### Appendiceal metastases

**Fig. 5:**
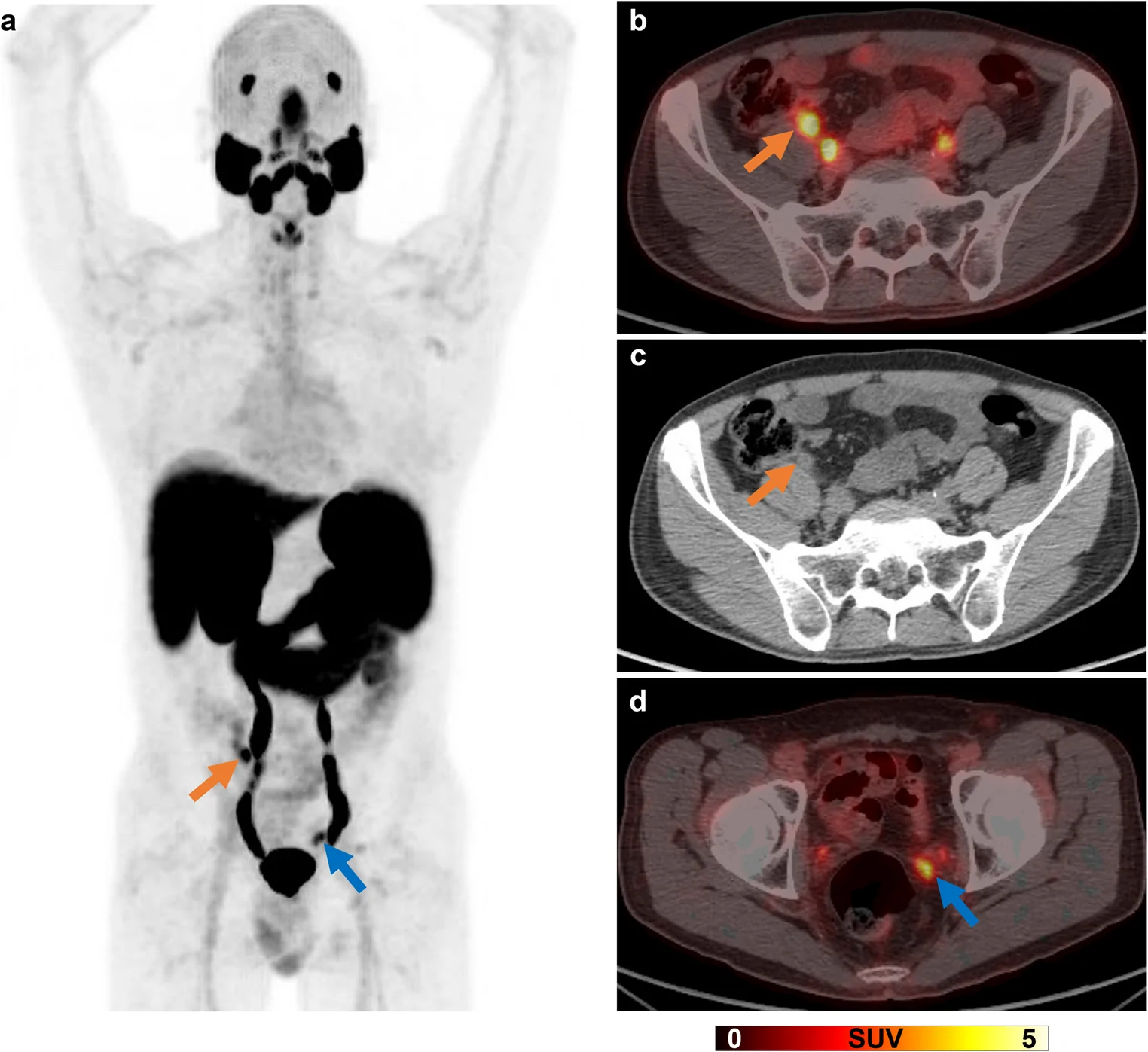
Patient with PSMA-avid appendiceal metastasis and left seminal vesicle bed recurrence. 56-year-old man with prostate cancer (Gleason 4+3) post-prostatectomy, ADT, and salvage RT, with rising PSA (0.8 ng/mL). (**a**) Maximum-intensity-projection (MIP) image showed focal uptake in the right lower quadrant (orange arrow). (**b**) Axial fused PET/CT and (**c**) axial CT image showed the uptake localized to the thickened appendix. Morphological appearance suggested appendicitis, and subsequent hemicolectomy confirmed appendiceal metastasis from prostate cancer. Uptake in left seminal vesicle bed (blue arrow) in (**a**) MIP and (**d**) fused PET/CT image, suspicious for tumor recurrence

Within the cohort, a subgroup of 4 patients who had appendiceal metastases was recognized. One was considered as either appendicitis per correlative CT or primary appendiceal tumor; he eventually underwent right hemicolectomy and was pathologically confirmed as metastatic prostate cancer (Fig. 5a, b, c, d). Three other patients had appendiceal and a few additional PSMA-avid peritoneal lesions that resolved/decreased on follow-up imaging under treatment with ADT and ARSI with decreased PSA.

## Discussion

Peritoneal metastases from prostate cancer, especially as the sole site of disease, are considered anecdotal with only a few case reports available in the literature. These are often regarded as a marker of aggressive cancer with a poor prognosis and might be overlooked on conventional imaging.

PSMA PET/CT has emerged as the most sensitive imaging modality for evaluating the extent of disease in prostate cancer [20]. Better sensitivity may allow detection of unusual sites of disease, such as the peritoneum, including as solitary sites of disease recurrence and in earlier stages, which may have significant implications on patient management.

The present retrospective study is the first to characterize patients with BCF and peritoneal metastases, detected by PSMA imaging. More than half of the patients in the present cohort were castrate-sensitive at the time of peritoneal disease detection and, in 33% of patients, the peritoneum was the only site of disease recurrence. Most patients had only a few peritoneal lesions. These findings may indicate that PSMA PET imaging is able to detect peritoneal metastases earlier in the course of disease. Moreover, 38% of patients had decreased or resolved disease on follow-up. Median OS was 53 months and patients who had only peritoneal metastases had 100% survival during follow-up. Better OS was associated with lower PSA values, lesser extent of peritoneal disease, and non-castrate resistance. These findings suggest that peritoneal metastases do not necessarily infer poor prognosis and are potentially treatable, possibly pointing toward a paradigm shift due to earlier detection and treatment.

PSMA PET/CT was able to detect peritoneal metastases even as an isolated finding, in small subcentimeter lesions, and in small quantities, demonstrating the high sensitivity of PSMA imaging. In comparison, FDG PET/CT and conventional CT/MRI may miss peritoneal metastases in a large proportion of patients. Given the rarity of peritoneal metastases in prostate cancer, management is not standardized. In the present cohort, most patients who responded to treatment were treated with ADT and ARPI. Previously, Delachambre et al. reported a case of a patient with isolated peritoneal metastases that had complete response to treatment with Abiraterone [19]. King et al. [11] reported good response to cytoreductive surgery and hyperthermic intraperitoneal chemotherapy. The authors also performed a literature review of 18 case reports and concluded that treatment options are limited, and prognosis is poor overall.

Only a few cases have so far reported on treatment with ^177^Lu-PSMA-617 RLT for patients with peritoneal metastases from prostate cancer. Van Golan et al. [8] reported on a patient with metastatic castration-resistant prostate cancer (mCRPC) and peritoneal metastases treated with four cycles of 7.4 GBq ^177^Lu-PSMA-I&T who had a marked decrease in both peritoneal metastases and PSA value. Gupta et al. [21] treated ten patients with mCRPC and visceral metastases with one cycle of ^177^Lu-PSMA-617 therapy, including one patient with peritoneal disease, who was reported to have 8 weeks of progression-free survival before being lost to follow-up. A subgroup of patients had been treated with ^177^Lu-PSMA-617 RLT at our institution. Despite being a small group, this is the largest described cohort of patients with peritoneal disease treated with ^177^Lu-PSMA-617 RLT to date. In our cohort, patients tolerated RLT well without any significant side effects, suggesting that this treatment is safe for patients with peritoneal metastases. Per our local protocol, patients with peritoneal disease are given a steroid taper during their radioligand treatments, which may account for the fact that none of the patients suffered from bowel obstruction. All patients unfortunately progressed during or at the end of their treatment protocol. This could suggest that peritoneal disease may be more resistant to RLT or may serve as a predictor of poor response to therapy, as was previously shown to be the case for visceral metastases [22, 23]; however, larger (and ideally prospective) studies are needed to draw conclusions. Of note, 3 of 5 patients who were treated at outside institutions with ^177^Lu-PSMA-617 reportedly had good response to treatment. All three were chemotherapy-naïve, which raises the possibility that these treatments were given off-label in an earlier stage of disease. Given the recent FDA approval of ^177^Lu-PSMA-617 in chemotherapy-naïve patients, based on the encouraging results of the PSMAfore trial [24], one might expect that patients with peritoneal metastases may be eligible for RLT earlier in the course of their disease, and may show better response.

During our case search we encountered several peritoneal findings that may mimic peritoneal metastases from prostate cancer. Readers should be aware of these pitfalls and use clinical history, correlative imaging findings, and assessment of tracer-avidity to correctly identify them. For example, such findings could include splenosis, which would be expected to be PSMA-avid in patients with a history of splenic rupture or surgery, inflammatory or cystic lesions that would typically be PSMA-non-avid and may have specific CT characteristics and relevant clinical history or metastases from other cancers that may be PSMA-avid, but often would show higher avidity with other tracers such as FDG or DOTATATE according to the characteristics of the primary tumor.

We also reviewed four cases of patients who had prostate cancer metastases to the appendix, a rare finding that has only been described once [25] but nevertheless should be recognized.

The present study has several limitations: first, its retrospective nature and the small size of the cohort, which are expected when investigating a rare phenomenon. In addition, not all cases were histologically confirmed, as biopsies of all lesions are not feasible, and follow-up imaging served as the best available alternative. Finally, the assumption that all 9,641 scans reported as negative for peritoneal metastasis were true negatives may have led to underestimation of the false-negative rate. The subgroup of seven patients who underwent RLT is too small to draw firm conclusions regarding treatment efficacy, and the apparent progression may reflect selection bias toward patients with more advanced disease.

## Conclusion

Peritoneal metastases in prostate cancer are rare, but can be detected with PSMA PET/CT, even in small or isolated lesions, and are often missed on FDG PET/CT and CT/MRI. i Peritoneal metastases do not necessarily predict poor prognosis. Therapy with ^177^Lu-PSMA was safe in these patients in the present cohort; however, larger prospective cohorts are needed to evaluate the role of ^177^Lu-PSMA in the treatment of peritoneal metastases.

## Supplementary Information

Below is the link to the electronic supplementary material.

Supplementary Material 1

## Data Availability

Not applicable.
