# Supplementary material for: Shining the PSMA spotlight on peritoneal metastases in prostate cancer
Source: Eur J Nucl Med Mol Imaging. 2025 Dec 11;53(6):3552–61. doi: 10.1007/s00259-025-07703-3 (PMC13498614; doi:10.1007/s00259-025-07703-3)
Supplement: Supplementary file 1 [file 259_2025_7703_MOESM1_ESM.docx]

**Supplemental Material for**

**Shining the PSMA Spotlight on Peritoneal Spread in Prostate Cancer**

**European Journal of Nuclear Medicine and Molecular Imaging**

Jonathan Kuten^1*^, Stephanie Chahwan^1^, Charlie White^2^, Audrey Mauguen^2^, Heiko Schöder^1^, Simone Krebs^1,3-5^

^1^Molecular Imaging and Therapy Service, Department of Radiology, Memorial Sloan Kettering Cancer Center, New York, NY

^2^Department of Epidemiology & Biostatistics, Memorial Sloan Kettering Cancer Center, New York, NY

^3^Department of Nuclear Medicine, The University of Texas MD Anderson Cancer Center, Houston, TX

^4^Department of Imaging Physics, The University of Texas MD Anderson Cancer Center, Houston, TX

^5^Institute of Data Science in Oncology, The University of Texas MD Anderson Cancer Center, Houston, TX

***Corresponding Author (and First Author):** Jonathan Kuten, MD, MHA, Molecular Imaging and Therapy Service, Department of Radiology, Memorial Sloan Kettering Cancer Center

1275 York Avenue, New York, NY 10065

Email: [kutenj@mskcc.or](mailto:kutenj@mskcc.or); Phone: 212-639-7998; Fax: 212-717-3263

**Imaging protocols**

**^68^Ga-PSMA-11, ^18^F-DCFPyL**:

**Radiotracer & uptake.** Patients received either ⁶⁸Ga-PSMA-11 ~5 mCi (≈185 MBq) or ¹⁸F-DCFPyL ~9 mCi (≈333 MBq). Imaging commenced ~60 minutes post-injection.

**Acquisition.** PET was acquired from vertex to mid-/upper thigh with the arms above the head. **Bed duration:** ~4 min/bed for ⁶⁸Ga-PSMA-11 and ~3 min/bed for ¹⁸F-DCFPyL.

**CT for attenuation/anatomic localization.** Low-dose helical CT: 120 kVp, ~70 mAs, 3.75-mm slice thickness.

**Reconstruction.** PET data were corrected for attenuation, scatter, and randoms and reconstructed on a 128 × 128 matrix (transaxial FOV ~70 cm; voxel size ~5.5 × 5.5 × 3.3 mm³) using OSEM (2 iterations, 16 subsets) with time-of-flight and point-spread-function modeling; a Gaussian 6.4-mm FWHM post-filter was applied.

**Contrast.** No oral or IV contrast was administered for PSMA PET/CT.

**^18^F-FDG (FDG) PET/CT**:

**Patient prep.** Patients fasted ≥6 hours; capillary/serum glucose ≤200 mg/dL prior to injection.

**Radiotracer & uptake.** ¹⁸F-FDG 12 mCi (≈444 MBq) (range 10–20 mCi / 370–740 MBq), with PET acquisition ~60 minutes post-injection.

**Acquisition.** PET from skull base to mid-/upper thigh, ~3 min/bed, arms above the head.

**CT for attenuation/anatomic localization.** Low-dose helical CT: 120 kVp, ~70 mAs, 3.75-mm slice thickness.

**Reconstruction.** Corrections for attenuation, scatter, and randoms; OSEM (2 iterations, 16 subsets) with time-of-flight and PSF modeling on a 128 × 128 matrix (voxel ~5.5 × 5.5 × 3.3 mm³); Gaussian 6.4-mm FWHM post-filter.

**Contrast.** Patients received 500 mL diluted oral iohexol (Omnipaque, 9 mg I/mL) ~30 minutes before scanning. No IV contrast was administered.

**CT**:

**Technique.** Contrast-enhanced CT of the abdomen and pelvis (with or without the chest) was performed on multi-detector scanners using a rotation time of 0.7 s, tube potential 120 kVp, and automatic tube-current modulation (~120–380 mA). Axial images were reconstructed at 5 mm slice thickness with coronal and sagittal reformations.

**Oral contrast.** Patients received 500 mL of diluted iohexol oral solution (~9 mg iodine/mL) 30 minutes before scanning.

**IV contrast.** 100 mL of iopamidol 300 mg I/mL (Isovue-300) was administered at 2.5 mL/s with a 70-second injection-to-scan delay to obtain portal-venous–phase images.

**MRI**:

**Pelvis / Prostate MRI:** (used when pelvic disease was the clinical correlate)

**Scanner & coil.** Exams were performed on 1.5T or 3T systems using a phased-array body coil.

**Sequences.**

- High-resolution T2-weighted fast spin-echo in axial, sagittal, and coronal-oblique planes aligned to the prostate.
- Diffusion-weighted imaging (DWI) with b = 0/800–1000 s/mm² and synthesized high-b (up to b = 2000 s/mm²); ADC maps generated.
- Pre- and post-contrast 3D T1-weighted Dixon-based spoiled gradient-echo (e.g., DISCO/LAVA) with and without fat suppression.
- Optional fat-suppressed T1 for hemorrhage or nodal evaluation.

**Contrast.** Gadoterate meglumine (Clariscan; 0.5 mmol/mL) at 0.1 mmol/kg (0.2 mL/kg) IV.
Antiperistaltic agent per institutional routine when not contraindicated.

**Abdomen–Pelvis MRI:**

**Scanner & coil.** 1.5T or 3T, phased-array body coil.

**Sequences.**

- Coronal and axial T2-weighted fast spin-echo (with and without fat suppression).
- Axial T1-weighted in- and opposed-phase (3D Dixon or dual-echo).
- Pre- and post-contrast 3D T1-weighted Dixon-based spoiled gradient-echo (e.g., DISCO/LAVA) for dynamic and portal-venous phases.
- DWI with b = 50 and 800 s/mm² (ADC maps), from diaphragm through pelvis.

**Contrast.** Gadoterate meglumine (Clariscan; 0.5 mmol/mL) at 0.1 mmol/kg (0.2 mL/kg) IV.

# **Supplemental table 1 – prior and current treatments**

| **Treatment** | **N = 57**^1^ |
| --- | --- |
| **Prior**  Prostatectomy | 38 (67%) |
| Prostate RT | 42 (74%) |
| Brachytherapy | 3 (5.3%) |
| ^177^Lu-PSMA-617 | 3 (5.3%) |
| Chemotherapy | 14 (25%) |
| PARPi | 3 (5.3%) |
| Cryotherapy | 2 (3.5%) |
| ARSI | 28 (49%) |
| ADT | 46 (81%) |
| **Current (at time of PSMA scan)**  ADT | 37 (65%) |
| ARSI | 18 (32%) |
| PARPi | 0 (0%) |
| Chemotherapy | 7 (12%) |
| ^177^Lu-PSMA-617 | 1 (1.8%) |
| ^1^n (%)  RT – Radiation Therapy; ADT – Androgen Deprivation Therapy; ARSI - Androgen Receptor Signaling Inhibitors; PARPi – Poly(ADP-ribose) Polymerase Inhibitors | |
